# Prevalence and antibiotic susceptibility pattern of CTX-M type extended-spectrum β-lactamases among clinical isolates of gram-negative bacilli in Jimma, Ethiopia

**DOI:** 10.1186/s12879-018-3436-7

**Published:** 2018-10-20

**Authors:** Ahmed Zeynudin, Michael Pritsch, Sören Schubert, Maxim Messerer, Gabriele Liegl, Michael Hoelscher, Tefara Belachew, Andreas Wieser

**Affiliations:** 1 Chair of Medical Microbiology and Hospital Epidemiology, Max von Pettenkofer Institute, Faculty of Medicine, LMU Munich, Marchioninistr. 17, 81377 Munich, Germany; 2 Institute of Health Sciences, Jimma University, Jimma, Ethiopia; 3 Center for International Health (CIH), University of Munich (LMU), 80802 Munich, Germany; 4 Division of Infectious Diseases and Tropical Medicine, Medical Center of the University of Munich (LMU), 80802 Munich, Germany; 5 German Center for Infection Research (DZIF), Partner Site Munich, 80802 Munich, Germany; 6 Plant Genome and Systems Biology, Helmholtz Center Munich, German Research Center for Environmental Health, 85764 Neuherberg, Germany

**Keywords:** Gram-negative bacilli, Extended-spectrum beta-lactamase, CTX-M, Antimicrobial susceptibility, Ethiopia

## Abstract

**Background:**

The prevalence of extended-spectrum β-lactamases (ESBLs) have been reported in clinical isolates obtained from various hospitals in Ethiopia. However, there is no data on the prevalence and antibiotic susceptibility patterns of CTX-M type ESBL produced by Gram-negative bacilli. The aim of this study was to determine the frequency and distribution of the *bla*_CTX-M_ genes and the susceptibility patterns in ESBL producing clinical isolates of Gram-negative bacilli in Jimma University Specialized Hospital (JUSH), southwest Ethiopia.

**Methods:**

A total of 224 non-duplicate and pure isolates obtained from clinically apparent infections, were included in the study. Identification of the isolates was performed by MALDI-TOF mass spectrometry. Susceptibility testing and ESBL detection was performed using VITEK® 2, according to EUCAST v4.0 guidelines. Genotypic analysis was performed using Check-MDR CT103 Microarrays.

**Results:**

Of the total 112 (50.0%) isolates screen positive for ESBLs, 63.4% (71/112) tested positive for ESBL encoding genes by Check-MDR array, which corresponds to 91.8% (67/73) of the total *Enterobacteriaceae* and 10.3% (4/39) of nonfermenting Gram-negative bacilli. Among the total ESBL gene positive isolates, 95.8% (68/71) carried *bla*_CTX-M_ genes with CTX-M group 1 type15 being predominant (66/68; 97.1% of CTX-M genes). The *bla*_CTX-M_ carrying *Enterobacteriaceae* (*n* = 64) isolates showed no resistance against imipenem and meropenem and a moderate resistance rate against tigecycline (14.1%), fosfomycin (10.9%) and amikacin (1.6%) suggesting the effectiveness of these antibiotics against most isolates. On the other hand, all the *bla*_CTX-M_ positive *Enterobacteriaceae* showed a multidrug resistant (MDR) phenotype with remarkable co-resistances (non-susceptibility rates) to aminoglycosides (92.2%), fluoroquinolones (78.1%) and trimethoprim/sulfamethoxazol (92.2%).

**Conclusions:**

This study demonstrates a remarkably high prevalence of *bla*_CTX-M_ genes among ESBL-producing isolates. The high level of resistance to β-lactam and non-β-lactam antibiotics as well as the trend to a MDR profile associated with the *bla*_CTX-M_ genes are alarming and emphasize the need for routine diagnostic antimicrobial susceptibility testing for appropriate choice of antimicrobial therapy.

**Electronic supplementary material:**

The online version of this article (10.1186/s12879-018-3436-7) contains supplementary material, which is available to authorized users.

## Background

Extended-spectrum β-lactamases (ESBLs) are a predominant cause of β-lactam resistance in Gram-negative bacilli (GNB) [1, 2]. Incidences of infections caused by ESBLs producing GNB are increasing in prevalence worldwide, both in the healthcare as well as community settings, posing significant therapeutic challenges [3–5]. ESBLs are most often a plasmid mediated heterogeneous group of β-lactamase enzymes, that confer resistance to a wide range of commonly used β-lactam antibiotics including third generation cephalosporins (e.g., ceftriaxone, cefotaxime and ceftazidime) as well as monobactams (aztreonam) [6]. TEM and SHV type ESBLs used to be the dominant ESBL genotypes [7]. However, in the past decade, the CTX-M type ESBLs have become the most widely distributed and globally dominant genotypes [8].

The CTX-M type enzymes are a group of class A ESBLs that in general exhibit much higher levels of activity against cefotaxime and ceftriaxone than ceftazidime [6, 9]. The presence of CTX-M type ESBLs is often associated with co-resistance phenotypes in particular to fluoroquinolones and aminoglycosides, in addition to tetracycline, and trimethoprim/sulfamethoxazole co*-*resistance*,* which is commonly observed among TEM and SHV type ESBLs [10, 11]. The group of CTX-M type ESBLs currently constitutes more than 170 allelic variants, which cluster into five major groups based on sequence homologies. The five CTX-M groups are: CTX-M-1, CTX-M-2, CTX-M-8, CTX-M-9 and CTX-M-25 [12]. Each group consists of a number of particular variants with dominant variants being restricted in distribution to specific geographic areas, while few others are globally distributed. CTX-M-14 and CTX-M-15 were the most commonly isolated variants worldwide [10, 13].

In Africa, CTX-M-15 (of the CTX-M-1 group) is the most frequently reported variant, although some other variants were also detected in the region [14, 15]. CTX-M type ESBLs have now spread and could be detected among many different bacterial strains of clinical importance. This is particularly true for *Enterobacteriaceae* revealing an ESBL phenotype such as *Escherichia coli* and *Klebsiella pneumoniae*, which often cause potentially serious infections in the hospital as well as community setting [13].

In Ethiopia, multiple studies have reported prevalence of ESBLs ranging from 25 to 38.5% among *Enterobacteriaceae* in clinical samples obtained from various hospitals, including Jimma University Specialized Hospital (JUSH) [16–19]. However, there is no data on the prevalence and antibiotic susceptibility patterns of CTX-M type ESBLs produced by GNB. Therefore, the aim of the present study was to determine the relative frequency and distribution of the *bla*_CTX-M_ genes, as well as the overall susceptibility patterns in ESBL producing clinical isolates of GNB in JUSH, southwest Ethiopia.

## Methods

### Study setting and clinical specimens

A total of 224 randomly selected, non-duplicate, pure and clinically relevant Gram-negative bacilli isolates recovered from various clinical specimens submitted to the bacteriology laboratory for routine culture and antimicrobial susceptibility testing at JUSH during March to October 2014 were included in the study. The isolates were stored in − 20 °C freezers until transport and subsequently shipped to the Department of Bacteriology, Max von Pettenkofer-Institute (LMU), Munich, Germany for further screening and molecular analysis. The specimens were sent from different inpatient and outpatient units of JUSH, the only teaching and referral hospital in the southwestern part of Ethiopia, providing health services for approximately 15 million people in the catchment area. The specimens included wound swabs, urine, biopsies, sputum and others (see Additional file 1). All inpatient clinical specimens were obtained after more than 48 h of hospitalization of the patient. Along with the specimens, basic demographic and medical data were recorded using standard clinical and laboratory record forms.

### Bacterial isolation, identification and susceptibility testing

Isolation and identification of the bacterial isolates was performed using standard microbiological techniques in use at the bacteriology laboratory in JUSH [20]. At the Max von Pettenkofer-Institute (LMU), all isolates were identified to the species level by MALDI-TOF mass spectrometry (MALDI Biotyper, Bruker Daltonik, Bremen, Germany, Biotyper software package, version 3.0) [21], and then retested for antibiotic susceptibilities using VITEK® 2 compact automated system (N215 and N248, bioMérieux, France), according to the instructions of the manufacturers. Software supplied by the manufacturer in compliance with the EUCAST v4.0 guidelines was used. The system included an Advanced Expert System (AES) that analysed growth patterns and detected the phenotype of organisms. Calculated MICs of piperacillin, piperacillin-tazobactam, cefotaxime, ceftazidime, cefepime, aztreonam, imipenem, meropenem, amikacin, gentamicin, ciprofloxacin, tobramycin, moxifloxacin, fosfomycin, tigecycline, colistin and trimethoprim/sulfamethoxazole were determined and interpreted according to EUCAST v4.0 guidelines [22].

### ESBL screening and phenotypic tests

All *Enterobacteriaceae* isolates with reduced susceptibility or resistance to ceftazidime and/or cefotaxime and/or aztreonam [23] and all non-fermenting GNB with multi-resistant phenotype [24] were considered as ESBL-screen positive and subjected to phenotypic and genotypic analysis. Phenotypic detection of ESBL production was performed with the VITEK® 2 compact automated systems (bioMérieux, France).

### Detection and molecular characterization of β-lactamase genes

Detection and molecular characterization of the β-lactamase genes was performed on all ESBL-screen positive isolates using Check-MDR CT103 Microarray Kits (Check-Points B.V., Wageningen, The Netherlands) following the manufacturer’s instructions. With this assay, mutation analysis of TEM and SHV genes was performed to separate wild type (WT) alleles from ESBL variants, further AmpC β-lactamases (CMY-I/MOX, ACC, DHA, ACT/MIR, CMY-II, FOX) and carbapenemases (KPC, NDM, VIM, IMP, OXA-48-like) were investigated. Finally, CTX-M group ESBLs 1, 2, 8 plus 25, and 9 are also detected with the chip. To further define the type of CTX-M group − 1 and − 9 genes specifically, all positive isolates were amplified with primers suggested by Kim et al. [25]. For CTX-M-1 group, the primers with the sequence 5-cgtcacgctgttgttaggaa-3 and 5-acggctttctgccttaggtt-3 were used at 55 °C annealing temperature to yield a 780 bp fragment. CTX-M-9 group genes were amplified with the primers 5-tattgggagtttgagatggt-3 and 5-tccttcaactcagcaaaagt-3 at 50 °C annealing temperature to yield a 932 bp fragment. The fragments were sequenced for allele type identification. In combination with the Check-MDR hybridization the CTX-M subtypes can thereby be identified with high confidence, although a theoretical uncertainty remains, as the gene is not completely covered by the sequencing.

### Quality control

For ESBL testing, *K. pneumoniae* ATCC 700603 (ESBL positive), *E. coli* CCUG62975 (ESBL positive), *E. coli* ATCC 25922 (ESBL negative) and *P. aeruginosa* (ATCC 27853) were used as quality control (QC) in all tests.

### Statistical analyses

Statistical significance for comparison of proportions was calculated by the chi-squared test using Statistical Package for Social Sciences (SPSS, version 23, SPSS, Chicago, IL, U.S.A.). A value of *P* < 0.05 was considered as statistically significant.

### Ethical considerations

The study was approved by Jimma University Ethical Review Board.

## Results

### Clinical bacterial isolates and specimens

Of the total 224 Gram-negative bacterial strains, 112 (50%) isolates were considered as screen positive for ESBLs. These isolates consisted of 73 *Enterobacteriaceae* (31 *Klebsiella pneumoniae,* 2 *Klebsiella oxytoca*, 14 *Enterobacter cloacae*, 13 *Escherichia coli, 5 Providencia stuartii, 4 Proteus mirabilis, 3 Morganella morganii,* and 1 *Escherichia hermanii)* and 39 non-fermenting Gram-negative bacilli (14 *Acinetobacter baumanii*, 2 *Acinetobacter pittii,* 1 *Acinetobacter haemolyticus*, 14 *Pseudomonas aeruginosa*, 3 *Alcaligenes faecalis*, 4 *Stenotrophomonas maltophilia* and 1 *Bordetella bronchiseptica*). The majority of these isolates was recovered from inpatients (83.9%, *n* = 94) mainly from surgical wards (60.6%, *n* = 57) followed by medical wards (21.3%, *n* = 20) and from two types of specimens; wound (54.5%, *n* = 61) and urine samples (26.8%, *n* = 30), which together account for 81.3% (*n* = 91) of the total isolates (see also Additional file 1). The total 112 screen positive isolates were collected from 100 patients; 90 (90%) of patients yielded one isolate for inclusion whereas ten (10%) patients yielded multiple species (eight patients with two species and two patients with three species).

### Phenotypic detection of ESBLs

Phenotypic ESBL production was observed in 62.5% (*n* = 70) of the total screen positive isolates (*n* = 112) using VITEK® 2 compact automated system (bioMérieux, France).

### Genotypic detection of ESBL encoding genes

Of the total 112 screen positive isolates, 63.4% (*n* = 71) were positive for ESBL encoding genes by Check-MDR array. This corresponds to 91.8% (67/73) of the total *Enterobacteriaceae* and 10.3% (4/39) of non-fermenting Gram-negative bacilli, namely 3 *P. aeruginosa* and 1 *A. faecalis* isolate. No ESBL alleles were detected among *Acinetobacter* spp.*, S. maltophilia* and *B. bronchiseptica* (Table 1). Specimen wise, 60.7% (*n* = 37) of isolates from wound samples, 63.3% (*n* = 19) from urine, 66.7% (*n* = 8) from biopsy samples and all the isolates obtained from sputum samples (*n* = 6) as well as eye discharge (*n* = 1) were positive for ESBL encoding genes. Among total inpatient (*n* = 94) and outpatient (*n* = 18) isolates, ESBL genes were detected in 68.1% and 38.9% of the isolates respectively. The comparison of the difference in proportion should be taken with caution as convenient sampling was used and most specimens were obtained from inpatients. Four patients had two different ESBL-positive isolates (*E. cloacae* and *K. pneumoniae* in two cases cases, *E. coli* and *M. morganii*, and *P. aeruginosa* and *A. faecalis* in one case each). One of the four patients had an SHV 238S + 240 K mutation bearing *E. cloacae* and a CTX-M-15 positive *K. pneumoniae* in the specimen, whereas the three other patients each had two different species each positive for CTX-M-15.

**Table 1:** Frequency, distribution and combinations of *bla* genes among screen and ESBL gene positive Gram-negative isolates

Screen positive species	Total ESBL		Among ESBL gene positive isolates							
			SHV E240K + G238S alone		CTX-M alone		CTX-M + SHV E240K + G238S		Total CTXM	
	n:	%	n:	%	n:	%	n:	%	n:	%
*E. coli* (*n* = 13)	13	100	0	0	12	92.3	1	7.7	13	100
*K. pneumoniae* (*n* = 31)	30	96.8	0	0	29	96.7	1	3.3	30	100
*E. cloacae (n = 14)*	12	85.7	3	25.0	9	75.0	0	0	9	75.0
other *Enterobacteriaceae*^a^ (*n* = 15)	12	80.0	0	0	12	100	0	0	12	100
*P. aeruginosa* (*n* = 14)	3	21.4	0	0	3	100	0	0	3	100
other Non-fermenters^b^ (*n* = 25)	1	4	0	0	1	100	0	0	1	100
Total (*n* = 112)	71	63.4	3	4.2	66	92.9	2	2.8	68	95.8

^a^includes 3 *M. morganii,* 4 *P. mirablis,* 5 *P. stuartii,* 2 *K. oxytoca* and 1 *E. hermanii*

^b^includes 17 *Acinetobacter* species (14 *A. baumanii,* 2 *A. pittii* and 1 *A. haemolyticus)*, 3 *A. faecalis,* 4 *S. maltophilia* and 1 *B. bronchiseptica*

### Frequency and distribution of *bla*_CTX-M_ genes

From a total of 71 isolates carrying ESBL encoding genes, 68 (95.8%) carried CTX-M genes either alone or in combination with SHV and/or TEM genes. Sixty-four out of 67 (95.5%) *Enterobacteriaceae* and all non-fermenting GNB (*n* = 4) which carried ESBL encoding genes, were positive for CTX-M genes. The remaining three isolates negative for CTX-M (4.2%) carried SHV-type ESBLs (G238S + E240K) genes and were found to be *E. cloacae* obtained from wound samples. All TEM and SHV β-lactam genes detected were wild type except five G238S + E240K SHV type ESBLs. Three of the five were detected in *E. cloacae* in combination with wild type TEM. The other two were found in one *E. coli* and *K. pneumoniae* isolate along with CTX-M genes (Table 1).

### Combinations of *bla*_CTX-M_ with other β-lactamase genes

Multiple β-lactamase genes in a single strain were observed in 83.1% (*n* = 59) of the total isolates carrying ESBL encoding genes. From a total of 68 CTX-M positive isolates, 12 (17.6%) harbored CTX-M alone. The remaining 56 (82.4%) isolates carried CTX-M in combination with wild type TEM and/or SHV (except two SHV E240K + G238S) in different frequencies, which is partly explained due to the general presence of β-lactamases in some strains e.g. in *Klebsiella* spp. (Table 1).

### Frequency and distribution of CTX-M groups and types

CTX-M group 1 was the most dominant CTX-M group detected in 66 of 68 CTX-M positive isolates (97.1%), either alone (*n* = 63, 92.6%) or in combination with other groups (*n* = 3, 4.5%). All CTX-M-1 genes were sequenced and all were found to be allele CTX-M-15. The remaining two (2.9%) CTX-M positive isolates carried CTX-M group 9 (Table 2) genes which upon sequencing were identified as allele CTX-M-24.

**Table 2:** Frequency and distribution of CTX-M groups among CTX-M positive Gram-negative bacilli isolates

CTX-M positive species	CTX-M groups (total)								CTX-M group combinations									
	CTX-M-1		CTX-M-2		CTX-M-8 + 25		CTX-M-9		CTX-M-1 alone		CTX-M-1 + 2		CTX-M-1 + 9		CTX-M-1 + 2 + 8 + 25		CTX-M-9 alone	
	n:	%	n:	%	n:	%	n:	%	n:	%	n:	%	n:	%	n:	%	n:	%
*E. coli* (*n* = 13)	12	92.3	0	0	0	0	2	15.4	11	84.6	0	0	1	7.7	0	0	1	7.7
*K. pneumoniae* (*n* = 30)	30	100	1	3.3	0	0	0	0	29	96.7	1	3.3	0	0	0	0	0	0
*E. cloacae (n = 9)*	9	100	0	0	0	0	0	0	9	100	0	0	0	0	0	0	0	0
Other *Enterobacteriaceae*^a^ (*n* = 12)	11	91.7	0	0	0	0	1	8.3	11	91.7	0	0	0	0	0	0	1	8.3
*P. aeruginosa* (*n* = 3)	3	100	1	33.3	1	33.3	0	0	2	66.7	0	0	0	0	1	33.3	0	0
Other Non-Fermenters^b^ (*n* = 1)	1	100	0	0	0	0	0	0	1	100	0	0	0	0	0	0	0	0
Total (*n* = 68)	66	97.1	2	2.9	1	1.5	3	4.4	63	92.6	1	1.5	1	1.5	1	1.5	2	2.9

^a^includes 3 *M. morganii,* 4 *P. mirablis,* 2 *P. stuartii,* 2 *K. oxytoca* and 1 *E. hermanii*

^b^includes 1 *A. faecalis*

### Antibiotic susceptibility pattern of CTX-M positive gram-negative bacilli isolates

The antibiotic susceptibility testing for CTX-M-positive *Enterobacteriaceae* isolates demonstrated a MIC in the respective susceptible range in < 2% of cases against cephalosporins according to EUCAST guidelines. Susceptibilities to carbapenems and a few other substances were found to be much higher. In terms of non-susceptibility, the highest level of antibiotic resistances was observed as expected against β-lactams such as piperacillin and cephalosporins, but also against trimethoprim-sulfamethoxazole (92.2%), gentamicin (89.1%), and quinolones (75%). No isolates showed full resistance to imipenem or meropenem, and only 3.1% and 1.6% tested intermediate for these substances, respectively (Table 3). One *E. coli* isolate tested positive for CTX-M-15 but was measured susceptible to third generation cephalosporins using VITEK 2 as well as disc diffusion tests.

**Table 3:** In vitro antimicrobial resistance pattern of CTX-M-positive Gram-negative isolates

Species	CTX-M positive isolate % resistance																
	PI	PIT	CTX	CAZ	CPM	AT	IMP	MRP	AK	HLG	TOB	CIP	MOX	FO	TGC	CL	COT
*E. coli* (*n* = 13)	100	30.8	92.3	92.3	92.3	92.3	0	0	7.7	76.9	76.9	92.3	84.6	7.7	0	7.7	84.6
*K. pneumoniae* (*n* = 30)	100	60	100	96.7	96.7	96.7	0	0	0	90	96.7	66.7	80	0	0	0	93.3
*E. cloacae* (*n* = 9)	100	0	100	100	100	100	0	0	0	88.9	88.9	22.2	77.8	0	0	0	100
*M. morganii* (*n* = 3)	100	0	100	100	100	100	0	0	0	100	100	66.7	100	100	IR	IR	100
*P. mirablis* (*n* = 4)	100	0	100	100	100	100	0	0	0	100	100	25	25	50	IR	IR	75
*P. stuartii* (*n* = 2)	100	0	100	100	100	100	0	0	0	IR	IR	50	50	50	IR	IR	100
*K. oxytoca* (*n* = 2)	100	50	100	100	100	100	0	0	0	100	100	0	50	0	0	0	100
*E. hermanii* (*n* = 1)	R	R	R	R	R	R	S	S	S	R	R	R	R	S	S	S	R
Total *Enterobacteriaceae* (*n* = 64)	100	35.9	98.4	96.9	96.9	96.9	0	0	1.6	89.1	92.2	59.4	75	10.9	14.1	15.6	92.2
*P. aeruginosa* (*n* = 3)	66.7	66.7	IR	33.3	66.7	66.7	0	0	33.3	66.7	66.7	100	100	100	IR	0	IR
*A. faecalis* (*n* = 1)	R	S	IE	IE	IE	R	S	S	S	S	S	IE	S	IE	IE	IE	IE
Total GNB (*n* = 68)	98.5	36.8	97.1	92.6	94.1	95.6	0	0	2.9	88.2	91.2	60.3	76.5	14.7	17.6	14.7	91.2

*Key*: *PI* piperacillin, *PIT* piperacillin/tazobactam, *CTX* cefotaxime, *CAZ* ceftazidime, *CPM* cefepime, *AT* aztreonam, *IMP* imipenem, *MRP* meropenem, *AK* amikacin, *HLG* gentamicin, *TOB* tobramycin, *CIP* ciprofloxacin, *MOX* moxifloxacin*, FO* fosfomycine, *TGC* tigecycline, *CL* colistin, *COT* trimethoprim/sulfamethoxazole

*n* number of isolates*, S* sensitive*, R* resistant*, IR* intrinsic resistance*, IE* insufficient evidence

### Co-resistance (co-non-susceptibility) to non-β-lactam antibiotics

**Fig. 1:**
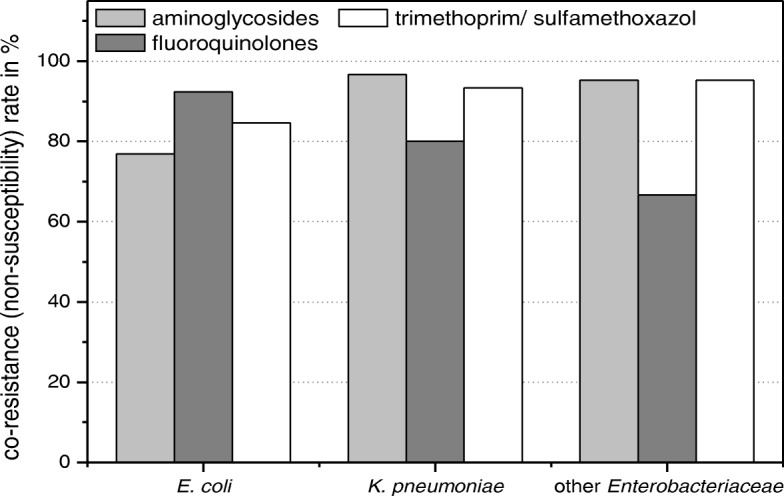
Bar graph showing the non-susceptibility pattern of the CTX-M positive *E. coli* (*n* = 13), *K. pneumoniae* (*n* = 30) and other *Enterobacteriaceae* (*n* = 21) against aminoglycosides, fluoroquinolones and trimethoprim-sulfamethoxazole

All the CTX-M-positive *Enterobacteriaceae* (*n* = 64, 100%) and *P. aeruginosa* (*n* = 3, 100%) were non-susceptible to ≥1 agent in ≥3 antimicrobial categories and hence defined as multidrug resistant (MDR) according to the international expert proposal for interim standard definitions for acquired resistance promoted by the European Centre for Disease Prevention and Control (ECDC) [26]. About 92.2%, 78.1% and 92.2% of the total CTX-M-positive *Enterobacteriaceae* were found to be non-susceptible (co-resistant) to aminoglycosides, fluoroquinolones and trimethoprim-sulfamethoxazole, respectively (Fig. 1).

### Non-susceptibility pattern in CTX-M and non*-*CTX-M carrying isolates

**Fig. 2:**
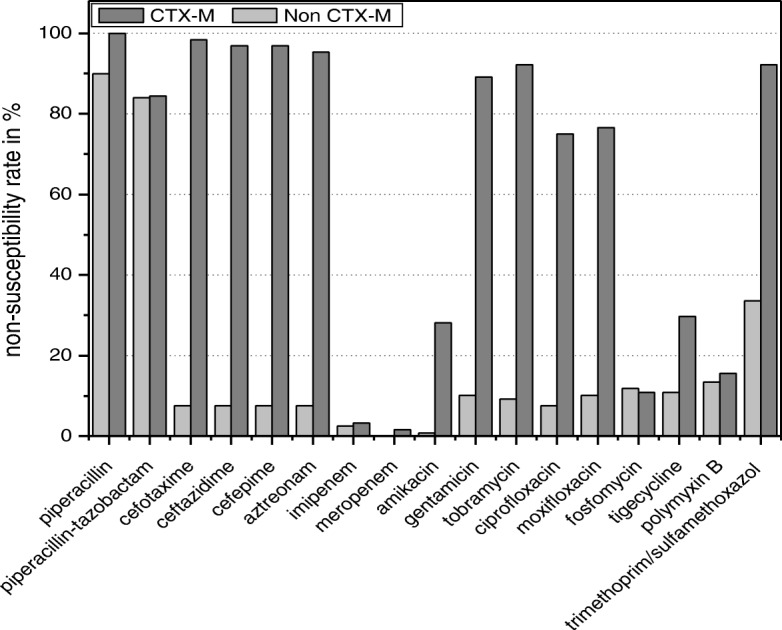
Comparison of non-susceptibility patterns of *bla*_CTX-M_ (*n* = 64) and non-*bla*_CTX-M_ (*n* = 119) *Enterobacteriaceae* isolates against the 17 different antibiotics tested

Both CTX-M (*n* = 64) and non-CTX-M-producing (*n* = 119) *Enterobacteriaceae* isolates have comparable non-susceptibility patterns to piperacillin/tazobactam, imipenem, meropenem, fosfomycin, and colistin/polymyxin B (*P* > 0.05). However, the non-susceptibility rate to all other antibiotics tested were all significantly higher among CTX-M-positive isolates compared to non*-*CTX-M ESBL-carrying isolates (*P* < 0.001) (Fig. 2). All the CTX-M negative isolates were also non-ESBLs except for three isolates expressing SHV type ESBLs. Unlike seen with CTX-M ESBLs, this did not affect the other non-susceptibilities.

## Discussion

The present study is the first report describing the molecular epidemiology of ESBL-encoding genes in Ethiopia. We demonstrate a high level of prevalence of CTX-M-type ESBLs among all ESBL positive isolates at JUSH. In total, 95.8% of all ESBL genes detected were of CTX-M type, and almost unanimously CTX-M-1 group variant type 15 (97.1% of all CTX-M positive isolates). These findings are in accordance with the fact that the CTX-M type ESBLs are the most widely distributed and globally dominant ESBL genotypes to date [13, 27, 28]. Of the groups, CTX-M-1 was also described to be highly prevalent in Italy [29], India [30], Switzerland [31], Saudi-Arabia [32], Syria [33], Pakistan [34] and China [35].

Factors and mechanisms which contribute to the emergence and increasing prevalence of CTX-M ESBLs of all groups are complex and may involve both, plasmid dissemination as well as clonal spread of bacterial strains [36, 37]. In addition, the selective pressure exerted by the frequent use of wide spectrum cephalosporins may promote their epidemiological success [10, 28, 38]. Especially in Ethiopia, the widespread misuse and overuse of cephalosporins may contribute to the selection and spread of CTX-M gene carrying clones [39–41]. The frequency of the CTX-M genotype among the ESBL gene-positive *Enterobacteriaceae* isolates was also remarkably high (95.5%) compared to similar findings among clinical *Enterobacteriaceae* isolates with prevalence rates of 91% in Brazil [42], 80.3% in Germany [43] and 79% in Switzerland [31].

Other than *E. coli* (92.3% CTX-M-15) and *K. pneumoniae* (100% CTX-M-15), CTX-M were also detected among other members of ESBL producing *Enterobacteriaceae (K. oxytoca, M. morganii, P. mirablis, P. stuartii, E. hermannii* and *E. cloacae)* as well as non-fermenting GNB *(P. aeruginosa* and *A. fecalis)* in 87.5% (*n* = 21) and 100% (*n* = 4), respectively. Out of all screen positive isolates (112) 41 were found to be non-ESBL producers. Thereby, most (35/41) were lactose non-fermenting GNB with known extensive intrinsic resistance mechanisms. Other isolates may be resistant due to genes not tested within this study, or due to derepression of wild type β-lactamases or even permeability defects. Among screen positive *Enterobacteriaceae* isolates, 92% (67/73) were also positive for an ESBL gene tested within this study.

Although, this study was small, it indicates the dissemination of the CTX-M genes to other GNB besides *Enterobacteriaceae* in Jimma. Similar findings have been reported in Switzerland [31], Argentina [44], Netherlands [45] and Japan [13]. The frequency of ESBL gene positive *Pseudomonas aeruginosa* was low (21.4%, *n* = 3) when compared to other GNB. This is probably due to the fact that most resistance mechanisms in *Pseudomonas aeruginosa* are mediated by the overproduction of AmpC β-lactamases as well as acquired metallo-β-lactamases, decreased permeability and efflux pumps [46]. In addition, plasmid incompatibility and host range of ESBL encoding plasmids might also play a role in our setting [13]. The emergence and spread of CTX-M-producing isolates in the community, particularly among *E. coli* in urinary tract infections (UTI), were reported from China [47], Brazil [48] and the UK [49]. A trend in this direction can also be seen in our study, as all the outpatient urine isolates of *E. coli* (*n* = 2)*, K. pneumoniae* (*n* = 2), *M. morganii* (*n* = 1), *P. mirablis* (*n* = 1) and *E. cloacae* (*n* = 1) with an ESBL gene were shown to carry CTX-M genes. However, the total sample size of outpatient isolates in the present study is small compared to the inpatient sample number.

The overall resistance pattern of the total CTX-M positive *Enterobacteriaceae* is very high for most antibiotics tested in the present study. The carbapenems (0% resistance) followed by amikacin (3% resistance) were found to have the highest susceptibility rates. However, all CTX-M-positive isolates identified in this study showed a MDR phenotype as well as remarkably high rates of co-resistance to fluoroquinolones, aminoglycosides, and trimethoprim/sulfamethoxazole. Only one *E. coli* isolate positive for an ESBL gene (CTX-M-15) was not resistant against third generation cephalosporins, while still maintaining an MDR phenotype. In this particular case, the CTX-M operon seems to be non-functional perhaps due to mutations. These findings are consistent with studies from Ghana [50], Lebanon [51] and India [52] which propose imipenem and amikacin as possible drugs for the management of infection caused by CTX-M-producing isolates. The results are also in accordance with findings of high prevalence of MDR phenotype (88.4%) among ESBL-producing *E. coli* and *K. pneumoniae* isolates in a previous phenotypic characterization of strains in JUSH [17]. Comparably high rates of co-resistance to non-β-lactam antibiotics were also reported from Brazil [42], South Korea [53] and Indian hospitals [54].

Surprisingly, colistin/polymyxin, which is not available in Ethiopia, showed resistance rates of above 10%. However, this rate has to be interpreted with caution, as the data based on VITEK® 2 testing system is unreliable for detecting colistin resistance [55], and results obtained by these methods may be overrated and require confirmation by ISO-standard broth microdilution method as nowadays recommended by EUCAST [56, 57]. As the respective recommendation was issued after completion of the study, it was not taken into consideration.

In the present study, only clinically relevant isolates of in- and outpatients were used, a screening upon admission, or screening of healthy controls was not performed. However, the high rates of ESBL positive organisms in outpatients without contact to the health care system within the last 3 months, argues for considerable ESBL carrier rates among the general population. Within the study population, mainly samples from internal medicine, pediatrics and ICU were ESBL positive and MDR, whereas in the surgical patient group many patients were found to harbor non-fermenters with MDR phenotype which are negative for the ESBL and carbapenemase genes tested within this study (see Additional file 2).

This conclusion is supported by a study conducted at black lion hospital in Addis Ababa (Ethiopia) reporting a high gastrointestinal colonization rate with ESBL producing *Enterobacteriaceae* among hospitalized patients [58]. It is well known, that many of the patients who develop health care-associated ESBL infections have preceding colonization of the gastrointestinal tract [59, 60]. A combination based on lack of hygiene and high colonization rates with ESBL positive organisms are likely to drive the ESBL rates in JUSH.

Within the sample group, other prominent resistance determinants were also investigated as part of the CT103 panel. Thereby, no KPC, NDM-1, VIM, IMP or Oxa48-like coding organism was detected. Previously, we could demonstrate the presence of NDM-1 in *Acinetobacter baumannii* in the area [61]. NDM-1 gene transfer to other isolates seems not to have occurred in relevant numbers. However, the presence of CTX-M-15 genes in different species in such high prevalence argues for horizontal gene transfer currently or in the past. The transfer might have occurred by plasmid exchange, which is especially common among *Enterobacteriaceae*, or by less frequent recombination events, e.g. involving IS elements. How recent or frequent such events have been cannot be elucidated given the methodology used, as the genes are found in numerous different species and isolates, it certainly cannot be explained simply by local clonal expansion of one strain.

## Conclusions

This study demonstrates a remarkably high level of CTX-M genes in GNB isolated in JUSH. The most predominant group was CTX-M-1 allele 15 and a few percent CTX-M-9 allele 24 among all the ESBLs gene positive clinical isolates. In South Africa, CTX-M-2 and -3 group are most prevalent, and CTX-M-14 and -15 in Egypt [62]. Meropenem, imipenem, colistin and amikacin were found to have the highest in vitro efficacy against the CTX-M-producing isolates. The high level of resistance to β-lactam and non-β-lactam antibiotics as well as the trend of a MDR profile associated with the CTX-M genes are alarming and emphasize the need for diagnostic antimicrobial susceptibility testing for appropriate choice of antimicrobial therapy and limiting the spread of antimicrobial resistance in Ethiopia and in the region.

## Additional files

Additional file 1: Distribution and frequency of GNB isolates in different clinical specimens. (PPTX 68 kb)

Additional file 2: Rates of ESBL and MDR in view of different hospital departments. (PPTX 44 kb)
