# Supplementary material for: Prevalence and antibiotic susceptibility pattern of CTX-M type extended-spectrum β-lactamases among clinical isolates of gram-negative bacilli in Jimma, Ethiopia
Source: BMC Infect Dis. 2018 Oct 20;18:524. doi: 10.1186/s12879-018-3436-7 (PMC6196031; doi:10.1186/s12879-018-3436-7)
Supplement: Supplementary file 1 — Distribution and frequency of GNB isolates in different clinical specimens. (PPTX 68 kb) [file 12879_2018_3436_MOESM1_ESM.pptx]

## Slide 1
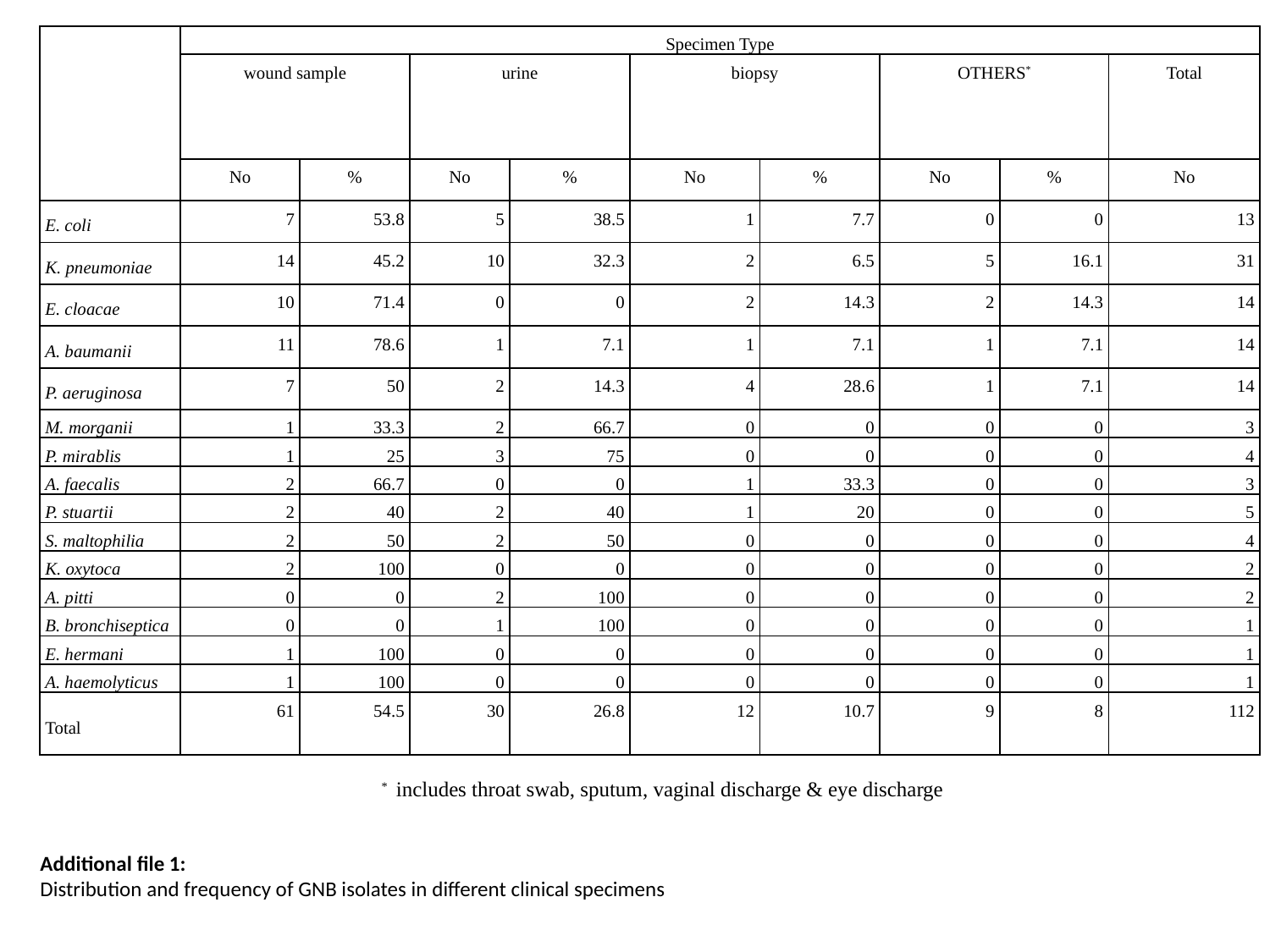

| | Specimen Type | | | | | | | | |
| --- | --- | --- | --- | --- | --- | --- | --- | --- | --- |
| | wound sample | | urine | | biopsy | | OTHERS\* | | Total |
| | No | % | No | % | No | % | No | % | No |
| E. coli | 7 | 53.8 | 5 | 38.5 | 1 | 7.7 | 0 | 0 | 13 |
| K. pneumoniae | 14 | 45.2 | 10 | 32.3 | 2 | 6.5 | 5 | 16.1 | 31 |
| E. cloacae | 10 | 71.4 | 0 | 0 | 2 | 14.3 | 2 | 14.3 | 14 |
| A. baumanii | 11 | 78.6 | 1 | 7.1 | 1 | 7.1 | 1 | 7.1 | 14 |
| P. aeruginosa | 7 | 50 | 2 | 14.3 | 4 | 28.6 | 1 | 7.1 | 14 |
| M. morganii | 1 | 33.3 | 2 | 66.7 | 0 | 0 | 0 | 0 | 3 |
| P. mirablis | 1 | 25 | 3 | 75 | 0 | 0 | 0 | 0 | 4 |
| A. faecalis | 2 | 66.7 | 0 | 0 | 1 | 33.3 | 0 | 0 | 3 |
| P. stuartii | 2 | 40 | 2 | 40 | 1 | 20 | 0 | 0 | 5 |
| S. maltophilia | 2 | 50 | 2 | 50 | 0 | 0 | 0 | 0 | 4 |
| K. oxytoca | 2 | 100 | 0 | 0 | 0 | 0 | 0 | 0 | 2 |
| A. pitti | 0 | 0 | 2 | 100 | 0 | 0 | 0 | 0 | 2 |
| B. bronchiseptica | 0 | 0 | 1 | 100 | 0 | 0 | 0 | 0 | 1 |
| E. hermani | 1 | 100 | 0 | 0 | 0 | 0 | 0 | 0 | 1 |
| A. haemolyticus | 1 | 100 | 0 | 0 | 0 | 0 | 0 | 0 | 1 |
| Total | 61 | 54.5 | 30 | 26.8 | 12 | 10.7 | 9 | 8 | 112 |
* includes throat swab, sputum, vaginal discharge & eye discharge
Additional file 1:
Distribution and frequency of GNB isolates in different clinical specimens
