# Supplementary material for: Prevalence and antibiotic susceptibility pattern of CTX-M type extended-spectrum β-lactamases among clinical isolates of gram-negative bacilli in Jimma, Ethiopia
Source: BMC Infect Dis. 2018 Oct 20;18:524. doi: 10.1186/s12879-018-3436-7 (PMC6196031; doi:10.1186/s12879-018-3436-7)
Supplement: Supplementary file 2 — Rates of ESBL and MDR in view of different hospital departments. (PPTX 44 kb) [file 12879_2018_3436_MOESM2_ESM.pptx]

## Slide 1
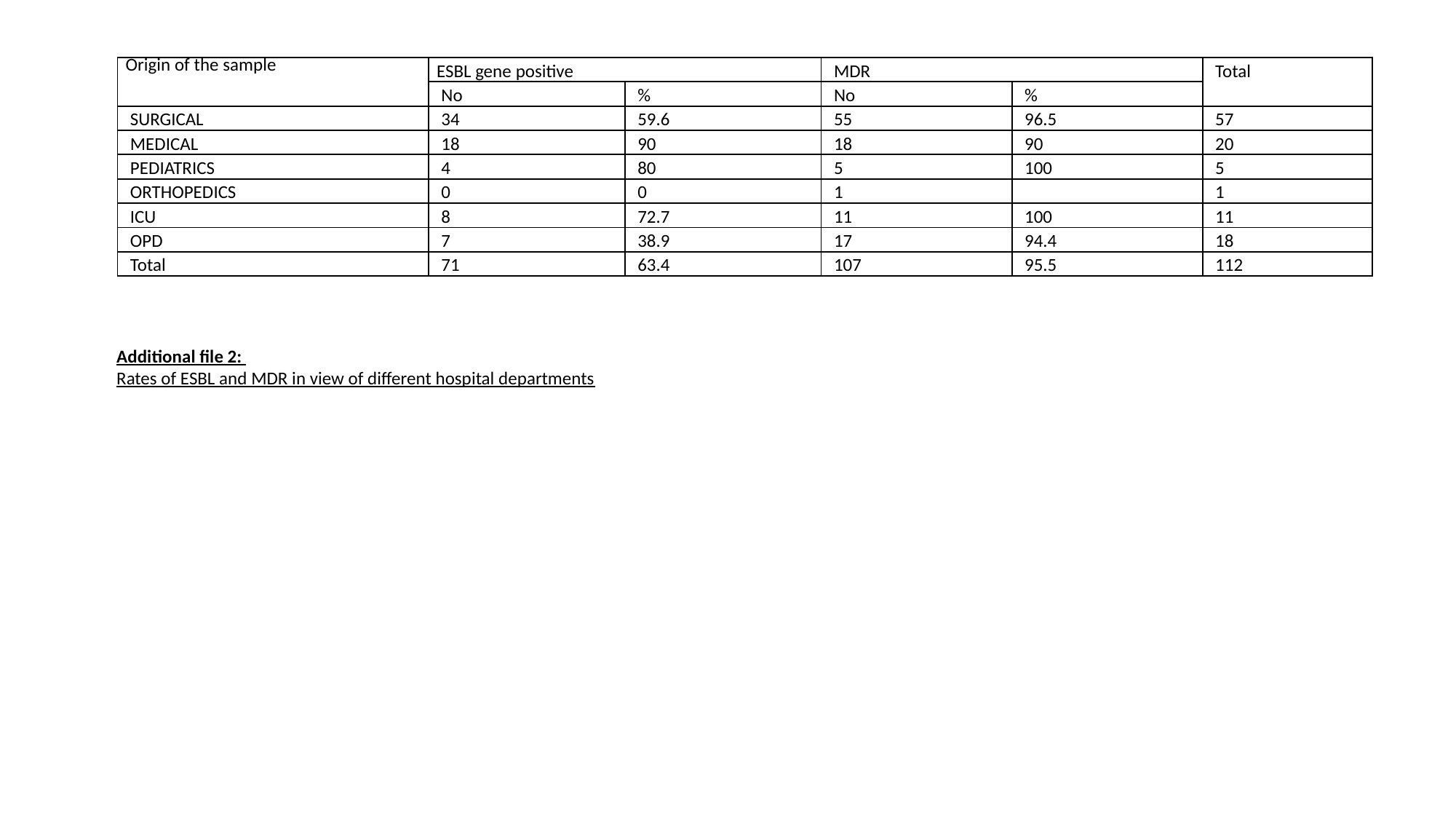

| Origin of the sample | ESBL gene positive | | MDR | | Total |
| --- | --- | --- | --- | --- | --- |
| | No | % | No | % | |
| SURGICAL | 34 | 59.6 | 55 | 96.5 | 57 |
| MEDICAL | 18 | 90 | 18 | 90 | 20 |
| PEDIATRICS | 4 | 80 | 5 | 100 | 5 |
| ORTHOPEDICS | 0 | 0 | 1 | | 1 |
| ICU | 8 | 72.7 | 11 | 100 | 11 |
| OPD | 7 | 38.9 | 17 | 94.4 | 18 |
| Total | 71 | 63.4 | 107 | 95.5 | 112 |
Additional file 2:
Rates of ESBL and MDR in view of different hospital departments
